# Analysis of the Effects of Sex Hormone Background on the Rat Choroid Plexus Transcriptome by cDNA Microarrays

**DOI:** 10.1371/journal.pone.0060199

**Published:** 2013-04-09

**Authors:** Telma Quintela, Isabel Gonçalves, Laura C. Carreto, Manuel A. S. Santos, Helena Marcelino, Filipa M. Patriarca, Cecília R. A. Santos

**Affiliations:** 1 CICS-UBI – Health Sciences Research Centre, University of Beira Interior, Covilhã, Portugal; 2 RNA Biology Laboratory, Department of Biology and CESAM, University of Aveiro, Aveiro, Portugal; Clermont Université, France

## Abstract

The choroid plexus (CP) are highly vascularized branched structures that protrude into the ventricles of the brain, and form a unique interface between the blood and the cerebrospinal fluid (CSF), the blood-CSF barrier, that are the main site of production and secretion of CSF. Sex hormones are widely recognized as neuroprotective agents against several neurodegenerative diseases, and the presence of sex hormones cognate receptors suggest that it may be a target for these hormones. In an effort to provide further insight into the neuroprotective mechanisms triggered by sex hormones we analyzed gene expression differences in the CP of female and male rats subjected to gonadectomy, using microarray technology. In gonadectomized female and male animals, 3045 genes were differentially expressed by 1.5-fold change, compared to sham controls. Analysis of the CP transcriptome showed that the top-five pathways significantly regulated by the sex hormone background are olfactory transduction, taste transduction, metabolism, steroid hormone biosynthesis and circadian rhythm pathways. These results represent the first overview of global expression changes in CP of female and male rats induced by gonadectomy and suggest that sex hormones are implicated in pathways with central roles in CP functions and CSF homeostasis.

## Introduction

The choroid plexus (CP), located in the cerebral ventricles, are branched and highly vascularized structures consisting of numerous villi, in which blood microvessels are enclosed by a single layer of cubical epithelial cells [1]. As a multipurpose organ, many functions have been attributed to CP, which depend primarily on the epithelial cells of this tissue. The best known function of CP is cerebrospinal fluid (CSF) formation, which not only regulates homeostasis in the central nervous system (CNS), providing buoyancy for the brain and spinal cord, but also participates in neural stem cell renewal, neuroprotection, sleep/awake cycles and in several neurological disorders [2]. CP is also known to participate in other functions that could be relevant in neurodegenerative diseases, as the control of the nutrient and hormone supply to the brain and CSF, the clearance of deleterious compounds and metabolites from the brain and the repair processes following brain damage [1], [3]. Moreover, the CP also participates in brain detoxification processes, neurohumoral brain modulation and neuroimmune interactions [3]. As a secretory engine, CP synthesizes biologically active substances, and recent studies demonstrated that these secretion might be delivered to the brain by encapsulated CP transplants, and minimize or prevent neural degeneration, particularly in models of stroke and Huntington's disease [4]. Moreover, the CP is also a target of centrally released transmitters [5]. In line with this multiplicity of functions, CP expresses a broad array of receptors and peptides; cytokines and cytokine receptors involved in the inflammatory processes, [6]; several receptors for neurotransmitters, growth factors and neuroprotective peptides [7]; sex hormone receptors, such as progesterone [8], estrogen receptors alpha and beta [9] and androgen receptors [10] suggesting that CP is also a target tissue for sex hormones. All the properties described above allow the CP to monitor the contents of the CNS extracellular fluid and respond to changes, for instance hormone alterations [11].

Recent studies provide insight into the neuroprotective effects of sex hormones and on their beneficial effects in neurodegenerative diseases [12], [13], particularly in Alzheimer's disease (AD), a pathology often associated with sexual hormone levels decline. In AD, CP synthesis, secretory, and transportation functions are significantly altered resulting in decreased CSF turnover [14]. The expression of some proteins including transthyretin [15], apolipoprotein J [16], gelsolin [17] and insulin growth factor 2 are also significantly decreased. In addition, some of these CP expressed proteins, such as beta-APP, glial cell line-derived neurotrophic factor, apolipoprotein E, brain derived neurotrophic factor [7] and transthyretin [18], [19], also implicated in neuroprotection and neurodegeneration, are estrogen and/or androgen responsive. Although many mRNA/proteins are differentially expressed in the CP in response to sex hormones, many others with distinct functions may be as well regulated by sex steroid hormones (SSHs) in CP with unknown consequences. Moreover, it is expected that increased knowledge about the physiology of CP will enable better understanding or even treating some brain diseases [20]. To gain additional insight into the functional role of SSHs in CP, the present study compared gene expression patterns between sham and gonadectomized (GDX) female/male rats CP and bring about new mechanisms and pathways potentially regulated by SSHs.

## Materials and Methods

### Ethics Statement

Animals were handled in compliance with the NIH guidelines and the European Union rules for the care and handling of laboratory animals (Directive 2010/63/EU). Animal experiments were also carried out according to the Portuguese law for animal welfare and the protocol was approved by the Committee on the Ethics of animal Experiments of the Health Science Research Centre of the University of Beira Interior (DGV/2011). Moreover, all efforts were made to minimize animal suffering.

### Animals and tissue collection

Wistar rats were housed under a 12-h light, 12-h dark cycle, with food and water *ad libitum* during the course of the experiment. Female and male rats (2 months±2 weeks) were either ovariectomized (OVX) or orchidectomized (OOX). Two weeks after surgery, castrated animals were anesthetized with a mixture of ketamine and medetomidine. Blood was collected to confirm hormone decline. Cerebral hemispheres were separated and both lateral ventricular CP removed and immediately frozen in liquid nitrogen. All samples were processed simultaneously to maximize the reproducibility of the results.

### Measurement of hormone levels

A blood sample was collected from the right atrium of the heart. Estradiol (E2) and testosterone (T) levels in plasma were measured by radioimmunoassays (RIAs) following the methodology described in [21]. Intra-assay and inter-assay precision (coefficient of variation) were 7.5% and 12.4% for T, and 6.9% and 8.3% for E2, respectively. The limit of detection of assays was 50 pg ml^−1^ for E2 and 200pg ml^−1^ for T. Statistical analysis was performed by Student's t-test and *p*-values were considered statistically significant at *p*<0.05.

### Microarray experimental design

For microarray analysis pools of 2 CP from each animal (each containing tissue removed from the lateral ventricles) were prepared. Total RNA was extracted using TRIzol reagent (Invitrogen, Carlsbad, CA, USA) following manufacturer's instructions. RNA was quantified using a NanoDrop spectrophotometer (NanoDrop Technologies), and RNA integrity was assessed by Bioanalyzer 2100 (Agilent Technologies, Santa Clara, CA, USA). All RNA samples used in this study had an A260/A280 ratio of>1.9. Pools of RNA samples from three animals were biological replicates. Equal amounts of RNA extract (200ng) from the 3 pools of sham or 3 pools of GDX animals, in a total of three biological replicates, were amplified and Cy-3-labeled using the Low Input Quick Amp Labeling kit (Agilent Technologies). Hybridizations were carried out on an Agilent-based microarray platform using custom-designed whole genome Rat GE 4x4K v3 microarrays, and arrays were scanned with an Agilent microarray scanner (Agilent Technologies). Scanned images were analyzed by Feature extraction software (Agilent Technologies) using GE1_105_Dec08 protocol. The signal intensity was normalized between microarrays by centering the median of the signal distribution using BRB-ArrayTools v3.8.1 (http://linus.nci.nih.gov/BRB-ArrayTools.html).

### Microarray data analysis

Differentially expressed genes were identified by pairwise comparison using a Student's t-test, with a *p*-value cut-off of 0.05. Only genes with a fold change above 1.5 were considered differentially expressed for further analyzes.

In order to identify pathways with a significant number of differentially expressed genes, the lists of differentially expressed genes from the female and male groups were imported into the database for Annotation, Visualization and Integrated Discovery (DAVID) v6.7 http://david.abcc.ncifcrf.gov/ [22]. Significantly enriched KEGG (Kyoto Encyclopedia of Genes and Genome http://www.genome.jp/kegg/pathway.html) pathways and Gene Ontology (GO) terms for Biological Process, Molecular Function and Cellular Component were identified using a *p*-value cut-off of 0.05. Redundant terms and categories comprising less than five genes were omitted.

### Real-Time qPCR validation

Quantitative Real-Time PCR was used to validate the microarray data for 15 selected target genes. Total RNA was prepared from lateral ventricular CP from the same pool of animals as outlined above. First-strand cDNA was synthesized using M-MLV Reverse Transcriptase (Invitrogen). Real-time qPCR was performed using SYBR Green assay and the iCycleriQ^TM^ system (Bio-Rad). Cycling parameters for all reactions were as follows: denaturation at 95°C for 10 min; 40 cycles of denaturation at 95°C for 15 sec and 1 min of annealing and extension at 60°C. Gene expression was determined relative to levels of expression of Cyclophilin A using the comparative Ct (2^−ΔΔCt^) method, with Ct denoting threshold cycle. Gene expression of Cyclophilin A has been shown not to change between sham and GDX animals. Standard deviations were calculated from triplicates of three separate samples from each group. For each sample, average Ct for each target gene was calculated as the mean of 3 technical replicates; ΔCt was calculated as the difference in average Ct of the target gene and the endogenous control gene. For each group, mean 2^−ΔCt^ was calculated as the geometric mean of 2^−ΔCt^ of the 3 samples of the group. Fold change was then calculated as mean 2^−ΔCt (GDX group)^/mean 2^−ΔCt (sham group)^. Fold change values above 1.0 indicate a positive expression or up-regulation relative to sham group. Fold change values below 1 indicate a negative expression or down-regulation relative to the sham group. Primers for target genes were designed using Primer-Blast-NCBI-NIH and are listed in Table 1. In addition, the resulting PCR products were run on a 1.5% agarose gel and bands were sequenced to verify the sequence identity.

**Table 1:** Primers used to validate microarray results by Real-Time PCR.

Gene name	Gene symbol	ID	Primer forward and reverse	Product size
Bcl2-like1	Bcl2l1	NM_001033670	TTCGGCACGAGCAGTCAGCC ACCAGCTCCCGGTTGCTCTGA	160bp
Mdm4 p53 binding protein homolog (mouse)	Mdm4	NM_001012026	CACGGTGCAACAGAGTGCTCC ACCAAGGCAGGCCAGCAACA	190bp
Insulin degrading enzyme	Ide	NM_013159	TGGCTGTGGACGCACCAAGGA GGGTGGCGCTTCGGAAAGGT	125bp
Nibrin	Nbn	NM_138873	AGTCATCCCCAGTGCGCCAA TCGGGGCCTTTCCCCTAACCA	233bp
Cullin 2	Cul2	NM_001108417	TGCTTCGGCACAACGCCCTC TGCTTGGCTGCGCTCGATGT	131bp
Spermidine synthase	Srm	NM_053464	GCGCTCGCGGTACCAAGACA TCAGCACCTTCCGCGGGTTG	164bp
Presenilin 1	PSen1	NM_019163.3	GAGGAAGACGAAGAGCTGACA GAAGCTGACTGACTTGATGGTG	114bp
Cystatin C	Cst3	NM_012837.1	TGGTGAGAGCTCGTAAGCAG GCTGGATTTTGTCAGGGTGT	203bp
Cathepsin D	Ctsd	NM_134334.2	GGCATGGGCTACCCTTTTAT GACAGCTCCCCGTGGTAGTA	182bp
Transferrin	Tf	NM_001013110.1	GCATCAGACTCCAGCATCAA CAGGACAGTCTGGTGCTTCA	312bp
Insulin-like growth factor 2	Igf2	NM_001190162.1	TGTCTACCTCTCAGGCCGTACTT GTGGCGCTTGGCCTCTCTGA	185bp
Cyclophilin A	Ppia	NM_017101.1	CAAGACTGAGTGGCTGGATGG GCCCGCAAGTCAAAGAAATTAGAG	163bp
Transthyretin	TTR	NM_012681.2	GGACTGATATTTGCGTCTGAAGC ACTTTCACGGCCACATCGAC	119bp
Beta-2 microglobulin	B2m	NM_012512.2	CCGTGATCTTTCTGGTGCTTGTC CTATCTGAGGTGGGTGGAACTGAG	150bp

## Results

### Characterization of the hormonal status of the animals

The efficacy of gonadectomy was confirmed by comparing serum E2 and T levels. In addition body weight gain and uterus weight of sham females were compared with OVX animals (Table 2). Serum E2 levels in ovariectomized (0.036±0.0036ng/mL) were significantly lower than in control animals (0.052±0.0034 ng/mL). Differences between OVX and sham animals in body weight gain (34.24±2.286 vs. 11.09±2.211 g/animal) and uterus weight (0.051±0.003 vs. 0.118±0.008 mg) reached statistical significance in both cases. In rat males, serum T levels decreased from an average of 2.625±0.445 ng/mL in sham animals to 0.238±0.0534ng/mL after surgery. No differences were found in body weight between OOX (38.02±3.736 g/animal) and sham animals (31.97±10.03 g/animal).

**Table 2:** Serum E2 and T levels, and body and uterus weight in rats at the time of sacrifice.

	Female Sham rats	Female ovariectomized rats	Male Sham rats	Male orchidectomized rats
No. of animals (n)	8	8	8	8
Change in serum E2 level (ng/mL)	0.052±0.0034	0.036±0.0036 **	-	-
Change in serum T level (ng/mL)	-	-	2.625±0.445	0.238±0.0534***
Change in body weight (g)	11.09±2.211	34.24±2.286***	31.97±10.03	38.02±3.736
Uterus weight (mg)	0.1184±0.008	0.051±0.003***	-	-

Values are means±SEM. Differences between gonadectomized and sham-operated rats were assessed using Mann-Whitney test: **p<0.001; ***p<0.0001.

### Genes differentially expressed in CP in response to gonadectomy

Microarray analysis of whole CP genome expression in OVX compared to sham female rats identified more than 6,000 (*p*-value<0.05) differentially expressed genes (approximately 25%). Differences in the transcriptome of OOX males compared with sham ranged approximately 15% of the rat CP transcriptome. Using a 1.5– fold change as a cut-off, 1168 genes (4.7%) were up-regulated in CP of female OVX rats, while in CP male rats only 426 genes (1.7%) were up-regulated (Table 3) relative to sham animals. The same trend can be seen in Table 3, for down-regulated genes, with 1328 genes (5.3%) being down-regulated in CP of female rats and 123 genes (0.5%) in CP of male rats.

**Table 3:** Number of up and down-regulated genes in rat CP in response to ovariectomy or orchidectomy.

Fold-change	Females No. of genes (% of genome)	Males No. of genes (% of genome)
Up-regulated		
>1.5	1168 (4.7%)	426 (1.7%)
>2.0	422 (1.7%)	156 (0.62%)
>5.0	11 (0.04%)	20 (0.08%)
Down- regulated		
>1.5	1328 (5.3%)	123 (0.5%)
>2.0	141 (0.56%)	36 (0.14%)
>5.0	29 (0.12%)	3 (0.012%)

To further understand the biological relevance of these gene expression profiles, we carried out bioinformatic analyses to determine the pathways and GO terms most significantly associated with these genes.

### Functional annotation analysis

In order to explore altered functional pathways in response to gonadectomy in female and male rat CP, we used the bioinformatics database DAVID. Pathway enrichment analysis was performed by comparing each list of differential expressed genes to all available biological pathways provided by KEGG. The subsequent KEGG-test analysis revealed 11 KEGG-pathways that were significantly changed (Table 4). The most affected pathway was olfactory transduction (102 up-regulated and 282 down-regulated genes in female rat CP and 42 up-regulated genes in male rats CP; Tables S1 and S2). Statistically significant changes were also found for genes involved in primary immunodeficiency, steroid hormone biosynthesis, taste transduction, maturity onset diabetes of the young, retinol metabolism, drug metabolism, metabolism of xenobiotics by cytochrome P450, pentose and glucuronate interconversions, ascorbate and aldarate metabolism and circadian rhythm (Table 4). Two of the pathways mentioned above, namely olfactory transduction and steroid hormone biosynthesis, were similarly regulated in female CP and in male rat CP. However, several pathways were exclusively up-regulated in female CP (Tables S1 and S7), such as primary immunodeficiency (7 genes; *p* = 8.5E-3), taste transduction (7 genes; *p* = 4.6E-2) and maturity onset diabetes of the young (6 genes; *p* = 1.1E-2). On the other hand, genes involved in retinol metabolism (18 genes; *p* = 6.4E-6), drug metabolism (16 genes; *p* = 1.1E-3), metabolism of xenobiotics by cytochrome P450 (15 genes; *p* = 4.9E-4), pentose and glucuronate interconversions (6 genes; *p* = 7.1E-3) and ascorbate and aldarate metabolism (6 genes; *p* = 1.3E-2), were significantly down-regulated only in female rat CP (Tables S1 and S7).

**Table 4:** KEGG pathways with significant association with genes differentially expressed in female and male rat choroid plexus.

	Females				Males			
	Up-regulated		Down-regulated		Up-regulated		Down-regulated	
Kegg pathways	Number of genes	P-value	Number of genes	P-value	Number of genes	P-value	Number of genes	P-value
Olfactory transduction	102	3.1E-12	282	1.7E-99	42	5.1E-5		
Primary immunodeficiency	7	8.5E-3						
Steroid hormone biosynthesis	7	2.6E-2	15	8.8E-6	5	1.7E-2		
Taste transduction	7	4.6E-2						
Maturity onset diabetes of the young	6	1.1E-2						
Retinol metabolism			18	6.4E-6				
Drug metabolism			16	1.1E-3				
Metabolism of xenobiotics by cytochrome P450			15	4.9E-4				
Pentose and glucuronate interconversions			6	7.1E-3				
Ascorbate and aldarate metabolism			6	1.3E-2				
Circadian rhythm							3	3.0E-3

Computational analysis allowed the detection of significantly enriched GO terms associated according to Biological Process, Molecular Function and Cellular Component.

**Figure 1:**
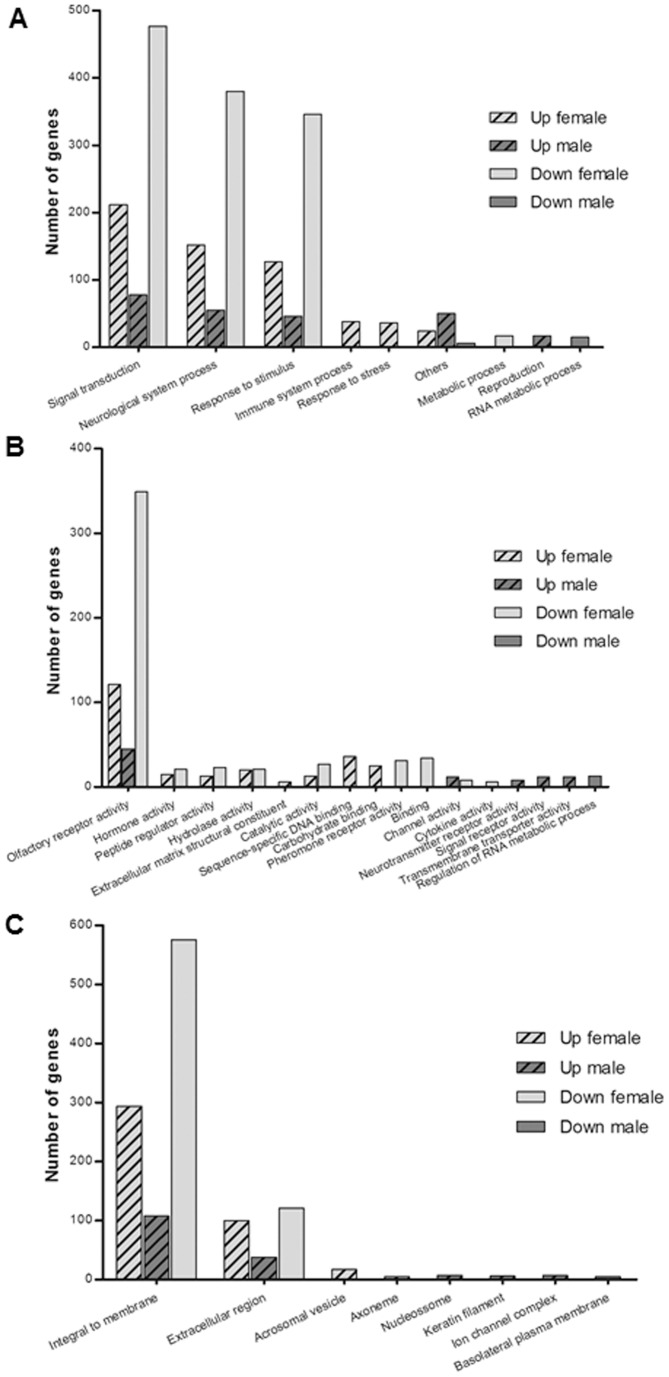
Gene ontology (GO) term enrichment analysis of differentially expressed genes. Biological processes (A), Molecular functions (B) and Cellular components (C).

Using DAVID analysis, the Biological Process domain of the Gene Ontology database provided the most extensive pathway assignments. Consistent with the pathway profiles, CP down-regulated male genes, were linked to biological processes (Fig. 1A) related to RNA metabolism (30 genes) and circadian rhythm process (6 genes) (Table S3). The majority of up- and down-regulated CP female genes and up-regulated CP male genes were related to signal transduction and response to stimulus (Fig. 1A). Interestingly, there was a massive up- and down-regulation of CP female genes related to the subcategories of cell surface receptor linked signal transduction and G-protein coupled receptor protein signaling pathways (Tables S4 and S5). The same was observed for genes up-regulated in CP of males (Tables S6 and S8). As shown in Tables S4, S5 and S6, the majority of differentially expressed genes corresponded to genes involved in the neurological system processes, cognition and sensory perception. In terms of response to stimulus, the most prominent processes affected were related to detection of chemical stimulus involved in sensory perception (Tables S4, S5 and S6). Regarding genes up-regulated in female rat CP, there was a significant overrepresentation in the subcategories of immune response (38 genes; *p* = 2.3E-3), defense response (36 genes; *p* = 4.1E-3) and acute inflammatory response (12 genes; *p* = 1.0E-2) (Table S4). Down-regulated genes in female rat CP were exclusively included in the categories of hormone metabolic process (17 genes; *p* = 5.7E-3) and retinoic acid metabolic process (7 genes; *p* = 6.4E-4), among others (Table S5). Male rat up-regulated genes related to reproduction were also significantly overrepresented (Table S6), indicating that a larger number of genes in this category increased in OOX animals.

Under the Molecular Function category (Fig. 1B) olfactory receptor activity, hormone activity, peptide regulator activity, hydroxylase activity and catalytic activity were overrepresented among the up- and down-regulated genes in female rat CP. Extracellular matrix structural constituents, sequence-specific DNA binding and carbohydrate binding proteins were exclusively up-regulated, whereas pheromone receptors, channels, cytokines and binding proteins were exclusively down-regulated in female rat CP. Olfactory receptors, channels, neurotransmitter receptors, signaling receptors and transmembrane transporters were overrepresented among the up-regulated genes in male rat CP, whereas regulation of RNA metabolism followed the opposite tendency.

Finally, under the Cellular Component category (Fig. 1C), integral to membrane and extracellular region were overrepresented among the up- and down-regulated genes in female rat CP and up-regulated genes in male rat CP. Acrossomal vesicle and axoneme were exclusively up-regulated in female rat CP. In addition, nucleossome, keratin filament and ion channel complex were exclusively up-regulated in male rat CP, whereas basolateral plasma membrane followed the opposite tendency.

### Validation of microarray results by quantitative Real-Time PCR analysis

To validate gene expression changes in rat CP induced by gonadectomy in the microarray analysis, qRT-PCR was performed in 15 genes that were either up or down-regulated in female or male GDX compared to sham animals. For 13 of these 15 genes there was complete agreement between the microarray results and the qRT-PCR results (Table 5). For almost all the cases the fold changes measured by qRT-PCR were less than those recorded using the microarray assay. For the two genes that were not consistent between the two methodologies, the microarray method gave an up-regulation of transcription, whereas the qRT-PCR method showed a down-regulation in the transcriptional response.

**Table 5:** Comparison of microarray and Real-Time PCR fold-changes of 15 selected genes differentially expressed in choroid plexus of gonadectomized female and male rats.

Gene	Gene name	Microarray	Real-Time PCR
Females			
Bcl2l1	Bcl2-like 1	1.61	0.59
Mdm4	Mdm4 p53 binding protein homolog (mouse)	1.39	0.96
Srm	Spermidine synthase	1.19	0.57
Ide	Insulin degrading enzyme	0.94	0.68
Nbn	Nibrin	0.81	0.48
Cul2	Cullin 2	0.83	0.62
Males			
Ide	Insulin degrading enzyme	1.10	1.33
Psen1	Presenilin 1	0.80	0.59
Bcl2l1	Bcl2-like 1	0.66	0.64
Cst3	Cystatin C	0.82	0.63
TTR	Transthyretin	0.81	0.95
Ctsd	Cathepsin D	0.79	0.94
Tf	Transferrin	0.81	0.76
Igf2	Insulin-like growth factor 2	0.81	0.93
B2m	Beta-2 microglobulin	0.86	0.74

## Discussion

CP is a brain structure involved in a variety of neurological disorders, including neurodegenerative, inflammatory, infectious, traumatic, neoplastic, and systemic diseases [20]. Moreover, the CP actively secretes CSF into the brain ventricles and creates the blood-CSF barrier. For this reason, CP epithelial cells may respond to factors released into the CSF following brain damage [2]. The CP has also been implicated in neuroendocrine signaling [23], amyloid clearance [24] and thyroid hormone distribution [25]. It has been demonstrated that CP also express sex hormone receptors, and synthesizes some peptides implicated in neuroprotection and neurodegeneration which are hormone responsive [26], [27]. Although there is evidence that the regulation of some of the molecules synthesized in CP, such as transthyretin, gelsolin and vasopressin [20] may have implications in the treatment of brain diseases, an analysis of the effects of SSHs on the transcriptome of female and male rat CP had never been done before.

To our knowledge, this study shows for the first time the response of the transcriptome of female and male rat CP to a decline in sex hormone levels induced by gonadectomy. The results presented here were validated by qRT-PCR, which confirmed the differential expression of selected candidate genes.

Compared to sham animals, gonadectomy generated more transcriptional changes in females than in males. As mentioned earlier in the results section, 2496 genes were differentially expressed more than 1.5-fold in CP from OVX compared to sham female rats.

KEGG functional pathway analysis in females revealed the enrichment of a variety of processes of CP physiology represented mostly by chemical senses pathways, such as the olfactory system and taste transduction and metabolic pathways, such as retinoic acid and xenobiotic detoxification pathways. It was recently reported that taste receptor genes Tas1r1, Tas1r2, Tas1r3, and their associated G-protein genes were detected in the intra-ventricular epithelial cells of CP, probably allowing neurons to have proximal access to the contents of the CSF and to modulate their responses [28]. Previous evidence on the regulation of this pathway by the hormonal background showed that female rats experience changes in taste bud morphology during pregnancy [29]. Moreover the response of the parabrachial pons to bitter taste is increased in OVX rats comparing to controls [30]. Hence, it seems likely that sex hormones affect taste and nutrient sensing both at the level of the taste buds as well as in the CNS [31]. In our study, declination of hormone levels clearly induced an up-regulation of 7 genes, including taste receptor genes and phospholipase C beta2, which were associated with taste transduction. Interestingly, in our study we identified differentially expressed receptors of the Tas2r family, important for the detection of bitter-tasting compounds [32]. Our comparative gene expression analysis provides the first evidence that the expression of taste-related genes in rat CP are under the control of SSHs. Finally, the fact that changes in expression levels occurred in CP suggests a role for this tissue as a taste sensor in the brain, most likely specialized in “tasting” the chemical composition of the CSF.

Hormone decline in rat CP had also important effects on other chemical sensing pathways, namely olfactory transduction signaling, with 102 genes being up-regulated and 282 genes being down-regulated. Among these differential expressed genes, most are olfactory receptors (ORs).There is evidence that SSHs trigger alterations in olfactory function in humans [33], and steroid hormones such as androstenone and androstadienone were identified as ORs ligands [34]. Initially identified in the sensory neurons of the olfactory epithelium, recent studies showed that different tissues throughout the body also expressed ORs [35], in different cell types, such as sperm, testis and kidney [36]. In these tissues their potential functions are unknown, but putative sensory roles have been proposed. Other interesting gene differentially expressed in rat female CP was phosducin. Phosducin is a protein highly abundant in the retinal photoreceptor cells and pinealocytes. However, higher levels of phosducin were also found in a small number of brain cells, such as habenular commissura, superior colliculus, ventral tegmental area and amygdala [37]. Together, our current transcriptome results suggest a possible function of CP in olfactory transduction, where OR genes may not function as odorant receptors but have other functions with important implications in the surveillance of the CSF composition. In fact, the expression of the olfactory signaling system has been previously found in the kidney and placenta [36], [38]. To our knowledge, this is the first report indicating the presence of the olfactory transduction machinery in CP, a barrier organ like the kidney and placenta.

Five metabolic pathways among the eleven pathways significantly modulated by hormone decline were: retinol metabolism, drug metabolism, metabolism of xenobiotics by cytochrome P450, pentose and glucuronate interconversions and ascorbate and aldarate metabolism. The overrepresentation of 18 significantly down-regulated genes in female CP transcriptome that were related to retinol metabolism, are in accordance with previous results described in other tissues [39], [40]. The components involved in retinoic acid homeostasis and in the metabolism and function of retinoids have been localized in cell populations of vascular and nervous tissues of the adult rat CNS [41], particularly in CP [42], [43]. Romand *et al.* revealed that retinol dehydrogenase (RDH10), shown to represent a limiting factor in the synthesis of retinoic acid, is present in the CP [44]. The down-regulation of RDH10 in female rat CP was demonstrated in our study, suggesting a possible role of SSHs in the production of retinaldehyde from retinol in CP that could be essential for the CNS development [44]. Moreover, several studies also suggest that, there will be different factors that regulate retinoic acid synthesis. Estrogens, for instance, coordinately up-regulate retinoic acid production and signaling in the human endometrium [39]. Furthermore, induction of the biosynthesis of retinoic acid occurs in the uterus of OVX rat, after estradiol administration [40]. Taken together, our current results together with the studies described above strongly favor the idea that SSHs, particularly estrogens, regulate CP retinol signaling.

We also confirmed the down-regulation of genes encoding molecules involved in the metabolism of drugs and xenobiotics. Long recognized for its great ability as a reabsorbing organ, the CP was once regarded as being primarily a tissue for clearing noxious substances [45], since the reabsorption of brain catabolites and drug metabolites by epithelial cells pivotally maintains CSF purity [46]. Alcohol dehydrogenase and cytochromes P450 are of particular interest in the metabolism of ethanol in the brain. In our study, we showed a down-regulation of alcohol dehydrogenase class IV (ADH4) and cytochromes P450 2 and 3 in female rat CP. Our results are in concordance with the study of Martinez *et al.*, who identified the presence of ADH1 and ADH4 in rat CP, suggesting a function of these enzymes as metabolic barriers [47]. Moreover, the rat CP has also been shown to be immunoreactive for Cyp1A1, an isoform involved in the metabolism of carcinogenic polycyclic aromatic hydrocarbons [48]. This isoform was identified in our microarrays results and is also down-regulated in female rat CP. It is interesting to note that steroid hormones contribute to the transcriptional regulation of enzymes that are involved in the metabolism of drugs and xenobiotics, such as P450s [49]. These observations are in agreement with our findings, suggesting that SSHs could exert a protective mechanism in CP, through the regulation of molecules involved in drug metabolism.

The identification of down-regulation of cytokine activity is consistent with the presence of cytokine receptors in CP epithelial cells [5]. In fact, CP secretes several inflammatory mediators that may change in response to specific stimuli [50]. Microarray analysis of the mouse CP gene regulation after inflammatory stimuli showed that the expression of several chemokines was altered [50]. Biological function annotation and enrichment showed the up and down-regulation of genes involved in a variety of processes, affecting several fields of the cell physiology, as signal transduction and response to stimulus. Other two well-represented subcategories such as immune system process and response to stress contain up-regulated genes whose functions may influence the interaction of molecules of the immune system with stress response. This hypothesis, if true, is in agreement with recent microarray observation indicating that chronic stress altered the rat CP expression of several genes, up-regulating genes involved in immune responses, such as, IL1b and TNF-α [51].

Enrichment analysis for cellular component and molecular function showed that the main enriched significantly up- and down-regulated genes found in these categories code for integral membrane proteins and also proteins found in the extracellular region with binding and catalytic activity. This finding corroborates the existence of a complex regulation of intracellular networks that control the catabolic processes triggered by SSHs upon reception of external stimulus from the peripheral circulation and/or the CSF.

Comparing CP gene expression from OOX with sham rat male, we have identified 549 genes differentially expressed 1.5-fold or above. When the up- and down-regulated genes were input into de KEGG pathway database, some clustering of transcriptional responses was evident, showing that orchidectomy induced differential expression of genes associated with steroid hormone biosynthesis and circadian rhythm.

In CP of rat males, orchidectomy clearly induced an up-regulation of steroid hormone biosynthesis, with five genes being up-regulated, such as Cytochrome P450 isoforms and 17-beta dehydrogenase 3. We have identified here, the presence of Cyp11b2, Cyp11b3 and of Cyp11b1 in rat CP. The later has been confirmed earlier by other authors [52]. These results suggest that circulating hormones could regulate the synthesis of mineralocorticoids and glucocorticoids in the CP and probably modulate CSF volume and electrolyte concentrations [52]. In the brain, emerging evidence indicates that, steroids directly synthesized within the CNS, either *de novo* from cholesterol or by *in situ* metabolism of circulating steroid precursors play an important role as endogenous modulators of neuronal functions and behavioral processes, and that alterations of neurosteroid concentrations may contribute to the pathophysiology of neuronal disorders [53]. Altogether, these findings suggest that CP may be a new steroidogenic organ responding to alterations in the blood and CSF hormonal composition through the activation of its own steroidogenic machinery.

Other interesting results were the decreased expression of three genes (basic helix-loop-helix family, period homolog 2 and 3), which integrate the mammalian circadian clock and play an important role in maintaining or organizing circadian rhythm [54]. The results obtained in our study are in agreement with the description of a relationship between androgens and circadian timing system, in which androgens modulate the hypothalamic suprachiasmatic nucleus timekeeping in a dose-dependent manner [55]. Moreover, at the behavioral level, gonadectomy produces a dramatic loss of the evening activity in male mice, that could be restored by testosterone treatment, suggesting that androgenic hormones regulate circadian responses [56].

The up-regulated rat male CP transcriptome was enriched with genes that clustered in ontologies characteristic of transport activity and binding, which are in accordance with the subcategories of integral to membrane and ion channel complexes. Specifically, the enrichment of genes in Biological process: signal transduction and response to stimulus is indicative of the importance of SSHs in the perception of molecular signals and the regulation of cellular downstream processes in CP. Down-regulated rat male genes in CP transcriptome enriched cellular component for basolateral plasma membrane. Enriched molecular functions indicated that down-regulated genes encoded proteins that regulate channel and cytokine activity and RNA metabolic processes.

## Conclusions

This is, to our knowledge, the first study demonstrating transcriptional regulation of female and male rat CP genes in response to different hormonal backgrounds. The GDX female and male CP suffered changes in the expression of genes associated with several pathways. Although more pronounced in females than in males, most of these CP expression changes occurred in genes implicated in chemical sensing, metabolism, steroid hormone biosynthesis and circadian rhythm pathways, adding to CP other putative functions that account for the efficient regulation of the CNS homeostasis and CSF composition. Moreover, the results described herein underscore the importance of the SSHs in the regulation of target genes that may acquire relevance as new potential therapeutic targets or agents to prevent or delay neurological disorders, and their overall impact in CP functions.

This work serves as a starting point for further studies, in order to examine in detail the origin of the alterations observed and to validate the observed changes using complementary approaches to disclose the functional implications of our findings.

## Supporting Information

Table S1 **Female CP differentially expressed genes associated with KEGG pathway analysis using DAVID.**
(DOCX)

Table S2 **Male CP differentially expressed genes associated with KEGG pathway analysis using DAVID.**
(DOCX)

Table S3 **Grouping of 3738 genes down-regulated in male CP according to their participation in biological processes (p<0.05) using DAVID.**
(DOCX)

Table S4 **Grouping of 6270 genes up-regulated in female CP according to their participation in biological processes (p<0.05) using DAVID.**
(DOCX)

Table S5 **Grouping of 6270 genes down-regulated in female CP according to their participation in biological processes (p<0.05) using DAVID.**
(DOCX)

Table S6 **Grouping of 3738 genes up-regulated in male CP according to their participation in biological processes (p<0.05) using DAVID.**
(DOCX)

Table S7 **The top 20 up and down regulated genes in the CP of female rats.** Differential gene expression between sham and gonadectomized female rats'CP. Genes were ranked on their fold changes and the twenty with the highest or lowest fold changes are shown here.
(DOCX)

Table S8 **The top 20 up and down regulated genes in the CP of male rats.** Differential gene expression between sham and gonadectomized male rats' CP. The genes were ranked on their fold changes and the twenty with the highest or lowest fold changes are shown here.
(DOCX)
