# Supplementary material for: Analysis of the Effects of Sex Hormone Background on the Rat Choroid Plexus Transcriptome by cDNA Microarrays
Source: PLoS One. 2013 Apr 9;8(4):e60199. doi: 10.1371/journal.pone.0060199 (PMC3622009; doi:10.1371/journal.pone.0060199)
Supplement: Table S1 — Female CP differentially expressed genes associated with KEGG pathway analysis using DAVID. (DOCX) [file pone.0060199.s001.docx]

| Kegg Pathways | **CP Female up-regulated genes** | **CP Female down-regulated genes** |
| --- | --- | --- |
| **Olfactory transduction** | - Chloride channel calcium activated 4-like - Cyclic nucleotide gated channel alpha 3 - Cyclic nucleotide gated channel alpha 4 - Cyclic nucleotide gated channel beta 1 - Olfactory receptors | Table S1. Female CP differentially expressed genes associated with KEGG pathway analysis using DAVID.   - Phosducin - Chloride channel calcium activated 5 - Protein kinase, cGMP-dependent, type II - Olfactory receptors |
| **Primary immunodeficiency** | - B-cell linker - Bruton agammaglobulinemia tyrosine kinase - CD19 molecule - Cd4 molecule - DNA cross-link repair 1C, PSO2 homolog (S. cerevisiae) - Regulatory factor X, 5 (influences HLA class II expression) - Similar to tumor necrosis factor receptor superfamily, member 13b; tumor necrosis factor receptor superfamily, member 13B |  |
| **Steroid hormone biosynthesis** | - Cytochrome P450, 3a18 - Cytochrome P450, subfamily 11B, polypeptide 3 - Hydroxy-delta-5-steroid dehydrogenase, 3 beta- and steroid delta-isomerase 1; 3 beta-hydroxysteroid dehydrogenase/delta-5-delta-4 isomerase type II - Hydroxy-delta-5-steroid dehydrogenase, 3 beta- and steroid delta-isomerase 5; similar to 3 beta-hydroxysteroid dehydrogenase type 3 (3 beta-hydroxysteroid dehydrogenase type III) (3Beta-HSD III) (NADPH-dependent 3-beta-hydroxy-delta(5)-steroid dehydrogenase) (3-beta-hydroxy-5-ene steroid dehydrogenase) (Progesterone reductas... - Hydroxysteroid (17-beta) dehydrogenase 3 - Steroid-5-alpha-reductase, alpha polypeptide 2 (3-oxo-5 alpha-steroid delta 4-dehydrogenase alpha 2) - Sulfotransferase family, cytosolic, 2B, member 1 | - UDP glucuronosyltransferase 1 family, polypeptide A2; - UDP glucuronosyltransferase 1 family, polypeptide A5; - UDP glycosyltransferase 1 family polypeptide A3; - UDP glucuronosyltransferase 1 family, polypeptide A6; - UDP glucuronosyltransferase 1 family, polypeptide A9; - UDP glycosyltransferase 1 family, polypeptide A8; - UDP glucuronosyltransferase 1 family, polypeptide A7C; - UDP glucuronosyltransferase 1 family, polypeptide A1 RG Rattus norvegicus - UDP glucuronosyltransferase 2 family, polypeptide A3; similar to UDP glycosyltransferase 2 family, polypeptide B4 - UDP glucuronosyltransferase 2 family, polypeptide B17 - UDP glucuronosyltransferase 2 family, polypeptide B36 - UDP glucuronosyltransferase 2 family, polypeptide B5; similar to UDP-glucuronosyltransferase 2B2 precursor (UDPGT) (3-hydroxyandrogen specific) (UDPGTr-4) (RLUG23); - UDP-glucuronosyltransferase 2 family, member 37 - UDP glycosyltransferase 2 family, polypeptide B - aldo-keto reductase family 1, member C18 - aldo-keto reductase family 1, member D1 (delta 4-3-ketosteroid-5-beta-reductase) - cytochrome P450, family 1, subfamily a, polypeptide 1 - cytochrome P450, family 3, subfamily a, polypeptide 2 - cytochrome P450, family 3, subfamily a, polypeptide 23/polypeptide 1; cytochrome P450, family 3, subfamily a, polypeptide 73 - cytochrome P450, family 7, subfamily a, polypeptide 1 - hydroxy-delta-5-steroid dehydrogenase, 3 beta- and steroid delta-isomerase 1; 3 beta-hydroxysteroid dehydrogenase/delta-5-delta-4 isomerase type II - hydroxy-delta-5-steroid dehydrogenase, 3 beta- and steroid delta-isomerase 5; similar to 3 beta-hydroxysteroid dehydrogenase type 3 (3 beta-hydroxysteroid dehydrogenase type III) (3Beta-HSD III) (NADPH-dependent 3-beta-hydroxy-delta(5)-steroid dehydrogenase) (3-beta-hydroxy-5-ene steroid dehydrogenase) (Progesterone reductas... - sulfotransferase family 1E, estrogen-preferring, member 1; estrogen sulfotransferase |
| **Taste transduction** | - Guanine nucleotide binding protein (G protein), beta polypeptide 3 - Phospholipase C, beta 2 - Sodium channel, nonvoltage-gated 1 gamma - Taste receptor, type 2, member 124 - Taste receptor, type 2, member 134 - Taste receptor, type 2, member 137 - Taste receptor, type 2, member 40 |  |
| **Maturity onset diabetes of the young** | - Forkhead box A3 - Hepatocyte nuclear factor 4, alpha - Homeobox gene HB9 - Insulin 1 - Islet amyloid polypeptide - Pyruvate kinase, liver and RBC |  |
| **Retinol Metabolism** |  | - UDP glucuronosyltransferase 1 family, polypeptide A2; - UDP glucuronosyltransferase 1 family, polypeptide A5; - UDP glycosyltransferase 1 family polypeptide A3; - UDP glucuronosyltransferase 1 family, polypeptide A6; - UDP glucuronosyltransferase 1 family, polypeptide A9; - UDP glycosyltransferase 1 family, polypeptide A8; - UDP glucuronosyltransferase 1 family, polypeptide A7C; - UDP glucuronosyltransferase 1 family, polypeptide A1 - UDP glucuronosyltransferase 2 family, polypeptide A3; similar to UDP glycosyltransferase 2 family, polypeptide B4 - UDP glucuronosyltransferase 2 family, polypeptide B17 - UDP glucuronosyltransferase 2 family, polypeptide B36 - UDP glucuronosyltransferase 2 family, polypeptide B5; similar to UDP-glucuronosyltransferase 2B2 precursor (UDPGT) (3-hydroxyandrogen specific) (UDPGTr-4) (RLUG23); UDP-glucuronosyltransferase 2 family, member 37 - UDP glycosyltransferase 2 family, polypeptide B - alcohol dehydrogenase 7 (class IV), mu or sigma polypeptide - aldehyde dehydrogenase 1 family, member A1 - aldehyde dehydrogenase 1 family, member A2 - cytochrome P450 4X1; cytochrome P450, family 4, subfamily a, polypeptide 8 - cytochrome P450, family 1, subfamily a, polypeptide 1 - cytochrome P450, family 2, subfamily c, polypeptide 12 - cytochrome P450, family 3, subfamily a, polypeptide 2 - cytochrome P450, family 3, subfamily a, polypeptide 23/polypeptide 1; cytochrome P450, family 3, subfamily a, polypeptide 73 - dehydrogenase/reductase (SDR family) member 9 - retinol dehydrogenase 10 (all-trans) - retinol dehydrogenase 11 (all-trans/9-cis/11-cis) - similar to Cytochrome P450 2C24 (CYPIIC24) (P450-PROS2) |
| **Drug Metabolism** |  | - UDP glucuronosyltransferase 1 family, polypeptide A2; - UDP glucuronosyltransferase 1 family, polypeptide A5; - UDP glycosyltransferase 1 family polypeptide A3; - UDP glucuronosyltransferase 1 family, polypeptide A6; - UDP glucuronosyltransferase 1 family, polypeptide A9; - UDP glycosyltransferase 1 family, polypeptide A8; - UDP glucuronosyltransferase 1 family, polypeptide A7C; - UDP glucuronosyltransferase 1 family, polypeptide A1 - UDP glucuronosyltransferase 2 family, polypeptide A3; similar to UDP glycosyltransferase 2 family, polypeptide B4 - UDP glucuronosyltransferase 2 family, polypeptide B17 - UDP glucuronosyltransferase 2 family, polypeptide B36 - UDP glucuronosyltransferase 2 family, polypeptide B5; similar to UDP-glucuronosyltransferase 2B2 precursor (UDPGT) (3-hydroxyandrogen specific) (UDPGTr-4) (RLUG23); UDP-glucuronosyltransferase 2 family, member 37 - UDP glycosyltransferase 2 family, polypeptide B - alcohol dehydrogenase 7 (class IV), mu or sigma polypeptide - aldehyde oxidase 3-like 1 - cytochrome P450, family 2, subfamily c, polypeptide 12 - cytochrome P450, family 2, subfamily d, polypeptide 2 - cytochrome P450, family 3, subfamily a, polypeptide 2 - cytochrome P450, family 3, subfamily a, polypeptide 23/polypeptide 1; cytochrome P450, family 3, subfamily a, polypeptide 73 - glutathione S-transferase Yc2 subunit - glutathione S-transferase, mu 5 - microsomal glutathione S-transferase 2 - similar to Cytochrome P450 2C24 (CYPIIC24) (P450-PROS2) |
| **Metabolism of xenobiotics by cytochrome P450** |  | - UDP glucuronosyltransferase 1 family, polypeptide A2; - UDP glucuronosyltransferase 1 family, polypeptide A5; - UDP glycosyltransferase 1 family polypeptide A3; - UDP glucuronosyltransferase 1 family, polypeptide A6; - UDP glucuronosyltransferase 1 family, polypeptide A9; - UDP glycosyltransferase 1 family, polypeptide A8; - UDP glucuronosyltransferase 1 family, polypeptide A7C; - UDP glucuronosyltransferase 1 family, polypeptide A1 - UDP glucuronosyltransferase 2 family, polypeptide A3; similar to UDP glycosyltransferase 2 family, polypeptide B4 - UDP glucuronosyltransferase 2 family, polypeptide B17 - UDP glucuronosyltransferase 2 family, polypeptide B36 - UDP glucuronosyltransferase 2 family, polypeptide B5; similar to UDP-glucuronosyltransferase 2B2 precursor (UDPGT) (3-hydroxyandrogen specific) (UDPGTr-4) (RLUG23); UDP-glucuronosyltransferase 2 family, member 37 - UDP glycosyltransferase 2 family, polypeptide B - alcohol dehydrogenase 7 (class IV), mu or sigma polypeptide - cytochrome P450, family 1, subfamily a, polypeptide 1 - cytochrome P450, family 2, subfamily c, polypeptide 12 - cytochrome P450, family 3, subfamily a, polypeptide 2 - cytochrome P450, family 3, subfamily a, polypeptide 23/polypeptide 1; cytochrome P450, family 3, subfamily a, polypeptide 73 - glutathione S-transferase Yc2 subunit - glutathione S-transferase, mu 5 - microsomal glutathione S-transferase 2 - similar to Cytochrome P450 2C24 (CYPIIC24) (P450-PROS2) |
| **Pentose and glucuronate interconversions/ Ascorbate and aldarate metabolism** |  | - UDP glucuronosyltransferase 1 family, polypeptide A2; - UDP glucuronosyltransferase 1 family, polypeptide A5; - UDP glycosyltransferase 1 family polypeptide A3; - UDP glucuronosyltransferase 1 family, polypeptide A6; - UDP glucuronosyltransferase 1 family, polypeptide A9; - UDP glycosyltransferase 1 family, polypeptide A8; - UDP glucuronosyltransferase 1 family, polypeptide A7C; - UDP glucuronosyltransferase 1 family, polypeptide A1 - UDP glucuronosyltransferase 2 family, polypeptide A3; similar to UDP glycosyltransferase 2 family, polypeptide B4 - UDP glucuronosyltransferase 2 family, polypeptide B17 - UDP glucuronosyltransferase 2 family, polypeptide B36 - UDP glucuronosyltransferase 2 family, polypeptide B5; similar to UDP-glucuronosyltransferase 2B2 precursor (UDPGT) (3-hydroxyandrogen specific) (UDPGTr-4) (RLUG23); - UDP-glucuronosyltransferase 2 family, member 37 - UDP glycosyltransferase 2 family |
