# Supplementary material for: Analysis of the Effects of Sex Hormone Background on the Rat Choroid Plexus Transcriptome by cDNA Microarrays
Source: PLoS One. 2013 Apr 9;8(4):e60199. doi: 10.1371/journal.pone.0060199 (PMC3622009; doi:10.1371/journal.pone.0060199)
Supplement: Table S2 — Male CP differentially expressed genes associated with KEGG pathway analysis using DAVID. (DOCX) [file pone.0060199.s002.docx]

| Kegg Pathways | **CP male up-regulated genes** | **CP male down-regulated genes** |
| --- | --- | --- |
| **Olfactory transduction** | - cyclic nucleotide gated channel alpha 3 - cyclic nucleotide gated channel alpha 4 - guanylate cyclase activator 1B - olfactory receptors |  |
| **Steroid hormone biosynthesis** | - cytochrome P450, family 3, subfamily a, polypeptide 23/polypeptide 1; - cytochrome P450, family 3, subfamily a, polypeptide 73 - cytochrome P450, subfamily 11B, polypeptide 2 - cytochrome P450, subfamily 11B, polypeptide 3 - hydroxysteroid (17-beta) dehydrogenase 3 - sulfotransferase family 2A, dehydroepiandrosterone (DHEA)-preferring-like 1; sulfotransferase family 2A, - dehydroepiandrosterone (DHEA)-preferring, member 2 |  |
| **Circadian rhythm** |  | - basic helix-loop-helix family, member e41 - period homolog 2 (Drosophila) - period homolog 3 (Drosophila) |

Table S2. Male CP differentially expressed genes associated with KEGG pathway analysis using DAVID.
