# Supplementary material for: Analysis of the Effects of Sex Hormone Background on the Rat Choroid Plexus Transcriptome by cDNA Microarrays
Source: PLoS One. 2013 Apr 9;8(4):e60199. doi: 10.1371/journal.pone.0060199 (PMC3622009; doi:10.1371/journal.pone.0060199)
Supplement: Table S3 — Grouping of 3738 genes down-regulated in male CP according to their participation in biological processes (p<0.05) using DAVID. (DOCX) [file pone.0060199.s003.docx]

| **GO Biological processes** | **CP of sham male rats versus OOX male rats** | **Count** | **%** | **P-value** |
| --- | --- | --- | --- | --- |
| RNA metabolic process | GO:0006355: regulation of transcription, DNA-dependent | 15 | 12.2% | 2.9E-2 |
|  | GO:0051252: regulation of RNA metabolic process | 15 | 12.2% | 3.5E-2 |
| Others | GO:0048511: rhythmic process | 6 | 4.9% | 3.9E-3 |

Table S3. Grouping of 3738 genes down-regulated in male CP according to their participation in biological processes (p<0.05) using DAVID.
