# Supplementary material for: Analysis of the Effects of Sex Hormone Background on the Rat Choroid Plexus Transcriptome by cDNA Microarrays
Source: PLoS One. 2013 Apr 9;8(4):e60199. doi: 10.1371/journal.pone.0060199 (PMC3622009; doi:10.1371/journal.pone.0060199)
Supplement: Table S4 — Grouping of 6270 genes up-regulated in female CP according to their participation in biological processes (p<0.05) using DAVID. (DOCX) [file pone.0060199.s004.docx]

| **GO Biological processes** | **CP of sham female rats versus OVX female rats** | **Count** | **%** | **P-value** |
| --- | --- | --- | --- | --- |
| Signal transduction | GO:0007166: cell surface receptor linked signal transduction | 212 | 18.4% | 1.1E-10 |
|  | GO:0007186: G-protein coupled receptor protein signaling pathway | 189 | 16.4% | 5.2E-14 |
| Neurological system process | GO:0050877: neurological system process | 152 | 13.2% | 1.2E-7 |
|  | GO:0050890: cognition | 145 | 12.6% | 7.7E-10 |
|  | GO:0007600: sensory perception | 142 | 12.3% | 2.6E-11 |
|  | GO:0007606: sensory perception of chemical stimulus | 134 | 11.5% | 4.5E-13 |
|  | GO:0007608: sensory perception of smell | 125 | 10.8% | 3.0E-11 |
|  | GO:0050909: sensory perception of taste | 8 | 0.7% | 1.2E-2 |
| Response to stimulus | GO:0051606: detection of stimulus | 127 | 11.0% | 1.0E-10 |
|  | GO:0009593: detection of chemical stimulus | 123 | 10.6% | 9.2E-11 |
|  | GO:0050906: detection of stimulus involved in sensory perception | 123 | 10.6% | 1.5E-10 |
|  | GO:0050907: detection of chemical stimulus involved in sensory perception | 122 | 10.6% | 9.2E-11 |
|  | GO:0050911: detection of chemical stimulus involved in sensory perception of smell | 120 | 10.4% | 2.4E-10 |
| Immune system process | GO:0006955: immune response | 38 | 3.3% | 2.3E-3 |
| Response to stress | GO:0006952: defense response | 36 | 3.1% | 4.1E-3 |
|  | GO:0002526: acute inflammatory response | 12 | 1.0% | 1.0E-2 |
| Others | GO:0007369: gastrulation | 9 | 0.8% | 3.5E-2 |
|  | GO:0042476: odontogenesis | 8 | 0.7% | 2.7E-2 |
|  | GO:0042475: odontogenesis of dentine-containing tooth | 7 | 0.6% | 2.6E-2 |

Table S4. Grouping of 6270 genes up-regulated in female CP according to their participation in biological processes (p<0.05) using DAVID.
