# Supplementary material for: Analysis of the Effects of Sex Hormone Background on the Rat Choroid Plexus Transcriptome by cDNA Microarrays
Source: PLoS One. 2013 Apr 9;8(4):e60199. doi: 10.1371/journal.pone.0060199 (PMC3622009; doi:10.1371/journal.pone.0060199)
Supplement: Table S5 — Grouping of 6270 genes down-regulated in female CP according to their participation in biological processes (p<0.05) using DAVID. (DOCX) [file pone.0060199.s005.docx]

| **GO Biological processes** | **CP of sham female rats versus OVX female rats** | **Count** | **%** | **P-value** |
| --- | --- | --- | --- | --- |
| Signal transduction | GO:0007166: cell surface receptor linked signal transduction | 477 | 36.4% | 9.9E-108 |
|  | GO:0007186: G-protein coupled receptor protein signaling pathway | 453 | 34.6% | 1.1E-131 |
| Neurological system process | GO:0050877: neurological system process | 380 | 29.0% | 1.9E-94 |
|  | GO:0050890: cognition | 368 | 28.1% | 1.8E-105 |
|  | GO:0007600: sensory perception | 361 | 27.5% | 4.7E-111 |
|  | GO:0007606: sensory perception of chemical stimulus | 352 | 26.8% | 1.4E-123 |
|  | GO:0007608: sensory perception of smell | 342 | 26.1% | 1.0E-120 |
|  | GO:0050909: sensory perception of taste | 10 | 0.8% | 5.5E-3 |
| Response to stimulus | GO:0051606: detection of stimulus | 346 | 26.4% | 3.2E-118 |
|  | GO:0050906: detection of stimulus involved in sensory perception | 341 | 26.0% | 1.5E-119 |
|  | GO:0009593: detection of chemical stimulus | 340 | 25.9% | 7.7E-120 |
|  | GO:0050911: detection of chemical stimulus involved in sensory perception of smell | 339 | 25.9% | 9.0E-122 |
|  | GO:0050907: detection of chemical stimulus involved in sensory perception | 339 | 25.9% | 1.3E-120 |
| Metabolic process | GO:0042445: hormone metabolic process | 17 | 1.3% | 5.7E-3 |
|  | GO:0034754: cellular hormone metabolic process | 14 | 1.1% | 5.4E-4 |
|  | GO: 0042573: retinoic acid metabolic process | 7 | 0.5% | 6.4E-4 |
|  | GO:0006776: vitamin A metabolic process | 7 | 0.5% | 1.2E-2 |
|  | GO:0016101: diterpenoid metabolic process | 7 | 0.5% | 2.0E-2 |
|  | GO:0001523: retinoid metabolic process | 7 | 0.5% | 2.0E-2 |
|  | GO:0006721: terpenoid metabolic process | 7 | 0.5% | 3.1E-2 |
|  | GO:0006775: fat-soluble vitamin metabolic process | 7 | 0.5% | 4.5E-2 |
|  | GO:0008207: C21-steroid hormone metabolic process | 5 | 0.4% | 8.8E-3 |

Table S5. Grouping of 6270 genes down-regulated in female CP according to their participation in biological processes (p<0.05) using DAVID.
