# Supplementary material for: Analysis of the Effects of Sex Hormone Background on the Rat Choroid Plexus Transcriptome by cDNA Microarrays
Source: PLoS One. 2013 Apr 9;8(4):e60199. doi: 10.1371/journal.pone.0060199 (PMC3622009; doi:10.1371/journal.pone.0060199)
Supplement: Table S6 — Grouping of 3738 genes up-regulated in male CP according to their participation in biological processes (p<0.05) using DAVID. (DOCX) [file pone.0060199.s006.docx]

| **GO Biological processes** | **CP of sham male rats versus OOX male rats** | **Count** | **%** | **P-value** |
| --- | --- | --- | --- | --- |
| Signal transduction | GO:0007166: cell surface receptor linked signal transduction | 78 | 18.4% | 5.9E-5 |
|  | GO:0007186: G-protein coupled receptor protein signaling pathway | 68 | 16.0% | 1.2E-5 |
| Neurological system process | GO:0050877: neurological system process | 55 | 13.0% | 1.7E-3 |
|  | GO:0050890: cognition | 52 | 12.3% | 3.8E-4 |
|  | GO:0007600: sensory perception | 50 | 11.8% | 2.2E-4 |
|  | GO:0007606: sensory perception of chemical stimulus | 46 | 10.8% | 1.4E-4 |
|  | GO:0007608: sensory perception of smell | 46 | 10.8% | 5.6E-5 |
| Response to stimulus | GO:0051606: detection of stimulus | 46 | 10.8% | 1.5E-4 |
|  | GO:0050911: detection of chemical stimulus involved in sensory perception of smell | 45 | 10.6% | 6.5E-5 |
|  | GO:0050906: detection of chemical stimulus involved in sensory perception | 45 | 10.6% | 7.9E-5 |
|  | GO:0009593: detection of chemical stimulus | 45 | 10.6% | 1.0E-4 |
|  | GO:0050907: detection of stimulus involved in sensory perception | 45 | 10.6% | 1.2E-4 |
| Reproduction | GO:0048609: reproductive process in a multicellular organism | 17 | 4.0% | 1.5E-2 |
|  | GO:0032504: multicellular organism reproduction | 17 | 4.0% | 1.5E-2 |
|  | GO:0019953: sexual reproduction | 15 | 3.5% | 1.6E-2 |
|  | GO:0007276: gamete generation | 13 | 3.1% | 2.8E-2 |
|  | GO:0007283: spermatogenesis | 11 | 2.6% | 2.6E-2 |
|  | GO:0048232: male gamete generation | 11 | 2.6% | 2.6E-2 |
|  | GO:00610: reproductive cellular process | 10 | 2.4% | 8.0E-3 |
| Others | GO:0045944: positive regulation of transcription from RNA polymerase II promoter | 14 | 3.3% | 4.1E-2 |
|  | GO:0048511: rhythmic process | 9 | 2.1% | 1.3E-2 |
|  | GO:0015837: amine transport | 7 | 1.7% | 3.3E-2 |
|  | GO:0003044: regulation of systemic arterial blood pressure mediated by chemical signal | 5 | 1.2% | 2.7E-3 |
|  | GO:0003073: regulation of systemic arterial blood pressure | 5 | 1.2% | 1.0E-2 |
|  | GO:0007586: digestion | 5 | 1.2% | 1.5E-2 |
|  | GO:0007623:circadian rhythm | 5 | 1.2% | 3.4E-2 |

Table S6. Grouping of 3738 genes up-regulated in male CP according to their participation in biological processes (p<0.05) using DAVID.
