# Supplementary material for: Analysis of the Effects of Sex Hormone Background on the Rat Choroid Plexus Transcriptome by cDNA Microarrays
Source: PLoS One. 2013 Apr 9;8(4):e60199. doi: 10.1371/journal.pone.0060199 (PMC3622009; doi:10.1371/journal.pone.0060199)
Supplement: Table S7 — The top 20 up and down regulated genes in the CP of female rats. Differential gene expression between sham and gonadectomized female rats'CP. Genes were ranked on their fold changes and the twenty with the highest or lowest fold changes are shown here. (DOCX) [file pone.0060199.s007.docx]

| **Gene** | **Description** | **Chromosome** | **Fold Change** | ***p*-value** |
| --- | --- | --- | --- | --- |
| Per2 | period homolog 2 (Drosophila) | chr9 | 7,6016 | 1,9644E-04 |
| Cradd | CASP2 and RIPK1 domain containing adaptor with death domain | chr7 | 5,3515 | 7,2231E-04 |
| Pde6a | phosphodiesterase 6A, cGMP-specific, rod, alpha | chr18 | 4,9872 | 3,1969E-06 |
| Adra2c | adrenergic, alpha-2C-, receptor | chr14 | 4,8760 | 1,9143E-02 |
| Slc26a9 | solute carrier family 26, member 9 | chr13 | 4,7479 | 1,5465E-02 |
| Lilrb4 | leukocyte immunoglobulin-like receptor, subfamily B, member 4 | chr1 | 4,6064 | 2,9161E-03 |
| Cys1 | cystin 1 | chr6 | 4,5159 | 3,5926E-03 |
| Agbl4 | ATP/GTP binding protein-like 4 | chr5 | 4,4285 | 4,3255E-03 |
| Olr428 | olfactory receptor 428 | chr3 | 3,8960 | 4,2312E-03 |
| Hlf | hepatic leukemia factor | chr10 | 3,7888 | 3,7022E-03 |
| Olr1679 | olfactory receptor 1679 | chr20 | 3,7656 | 3,8237E-04 |
| Bin2a | beta-galactosidase-like protein | chr8 | 3,7378 | 1,6369E-02 |
| Avpr1b | arginine vasopressin receptor 1B | chr13 | 3,7205 | 2,5798E-02 |
| Taar7b | trace amine-associated receptor 7b | chr1 | 3,6562 | 8,9296E-04 |
| Dbp | D site of albumin promoter (albumin D-box) binding protein | chr1 | 3,5995 | 2,7251E-02 |
| Cenpf | centromere protein F | chr13 | 3,5970 | 2,5311E-03 |
| Ppcdc | phosphopantothenoylcysteine decarboxylase | chr8 | 3,5376 | 1,6340E-02 |
| Olr382 | olfactory receptor 382 | chr1 | 3,5186 | 3,2008E-02 |
| Nkx2-3 | NK2 transcription factor related, locus 3 (Drosophila) | chr1 | 3,5005 | 4,6635E-03 |
| Vom1r56 | vomeronasal 1 receptor 56 | chr1 | 3,4637 | 2,8040E-02 |
| Hoxa2 | homeo box A2 | chr4 | -38,8840 | 4,7488E-04 |
| Sfrp2 | secreted frizzled-related protein 2 | chr2 | -28,1488 | 6,1608E-04 |
| Kcnj14 | potassium inwardly-rectifying channel, subfamily J, member 14 | chr1 | -23,8010 | 7,2320E-05 |
| Dbh | dopamine beta-hydroxylase (dopamine beta-monooxygenase) | chr3 | -15,0976 | 1,6738E-03 |
| Gfra1 | GDNF family receptor alpha 1 | chr1 | -10,6074 | 2,3832E-04 |
| Impg1 | interphotoreceptor matrix proteoglycan 1 | chr8 | -9,7631 | 1,9795E-02 |
| Mme | membrane metallo-endopeptidase | chr2 | -8,2876 | 2,5527E-04 |
| Crhbp | corticotropin releasing hormone binding protein | chr2 | -6,0530 | 2,1094E-02 |
| Arntl | aryl hydrocarbon receptor nuclear translocator-like | chr1 | -5,8312 | 4,7296E-03 |
| Il22 | interleukin 22 | chr7 | -5,7744 | 9,5668E-03 |
| Mptx | mucosal pentraxin | chr13 | -5,4069 | 5,9097E-04 |
| Batf3 | basic leucine zipper transcription factor, ATF-like 3 | chr13 | -5,1225 | 9,0974E-04 |
| Moxd1 | monooxygenase, DBH-like 1 | chr1 | -5,0422 | 6,0540E-06 |
| Tmem90b | transmembrane protein 90B | chr3 | -4,9945 | 1,1205E-02 |
| Gabra1 | gamma-aminobutyric acid (GABA) A receptor, alpha 1 | chr10 | -4,8539 | 1,7535E-02 |
| Irx2 | iroquois homeobox 2 | chr17 | -4,6123 | 7,7370E-03 |
| Wif1 | Wnt inhibitory factor 1 | chr7 | -4,4556 | 1,6855E-02 |
| Ceacam3 | carcinoembryonic antigen-related cell adhesion molecule 3 | chr1 | -4,2575 | 3,5881E-02 |
| Cts8 | cathepsin 8 | chr17 | -4,2555 | 2,4206E-02 |
| Fam3d | family with sequence similarity 3, member D | chr15 | -4,2258 | 1,1257E-03 |

Table S7. The top 20 up and top 20 down regulated genes in rat female CP

| Differential gene expression between sham and gonadectomized rat female CP. |
| --- |
| The genes were ranked on their fold changes and the twenty with the highest or lowest fold changes are shown here. |
