# Supplementary material for: Analysis of the Effects of Sex Hormone Background on the Rat Choroid Plexus Transcriptome by cDNA Microarrays
Source: PLoS One. 2013 Apr 9;8(4):e60199. doi: 10.1371/journal.pone.0060199 (PMC3622009; doi:10.1371/journal.pone.0060199)
Supplement: Table S8 — The top 20 up and down regulated genes in the CP of male rats. Differential gene expression between sham and gonadectomized male rats' CP. The genes were ranked on their fold changes and the twenty with the highest or lowest fold changes are shown here. (DOCX) [file pone.0060199.s008.docx]

| **Gene** | **Description** | **Chromosome** | **Fold change** | ***p*-value** |
| --- | --- | --- | --- | --- |
| Crisp2 | cysteine-rich secretory protein 2 | chr9 | 28,2411 | 2,5700E-05 |
| Tnp2 | transition protein 2 | chr10 | 27,1717 | 4,6100E-06 |
| Tnp1 | transition protein 1 | chr9 | 19,6352 | 9,9900E-05 |
| Smcp | sperm mitochondria-associated cysteine-rich protein | chr2 | 15,1353 | 1,9700E-05 |
| Gsg1 | germ cell associated 1 | chr4 | 14,9897 | 2,2509E-03 |
| Hmgb4 | high-mobility group box 4 | chr5 | 10,5736 | 5,2400E-05 |
| Gtsf1 | gametocyte specific factor 1 | chr7 | 9,0693 | 6,8600E-05 |
| Nt5c1b | 5'-nucleotidase, cytosolic IB | chr6 | 8,2799 | 2,2000E-05 |
| Spetex-2A | Spetex-2A protein | chr15 | 6,1889 | 2,0300E-04 |
| Hils1 | histone linker H1 domain, spermatid-specific 1 | chr10 | 5,0404 | 3,8031E-03 |
| Dspp | dentin sialophosphoprotein | chr14 | 4,8948 | 5,1806E-03 |
| Cmtm2a | CKLF-like MARVEL transmembrane domain containing 2A | chr19 | 4,8262 | 1,0147E-03 |
| Spetex-2G | Spetex-2G protein | chr15 | 4,7737 | 3,6500E-05 |
| Olr709 | olfactory receptor 709 | chr3 | 4,7470 | 5,6701E-03 |
| Art2 | ADP-ribosyltransferase 2 | chr1 | 4,4209 | 2,1273E-02 |
| Muc15 | mucin 15, cell surface associated | chr3 | 4,1992 | 9,7774E-03 |
| Arntl | aryl hydrocarbon receptor nuclear translocator-like | chr1 | 4,1832 | 1,2571E-03 |
| Il22 | interleukin 22 | chr7 | 3,8184 | 1,7980E-02 |
| Spt1 | salivary protein 1 | chr7 | 3,8007 | 4,9458E-02 |
| Iqcf5 | IQ motif containing F5 | chr8 | 3,6830 | 5,4500E-04 |
| Dbp | D site of albumin promoter (albumin D-box) binding protein | chr1 | -3,5960 | 4,1400E-05 |
| Kcnh5 | potassium voltage-gated channel, subfamily H (eag-related), member 5 | chr6 | -2,7935 | 3,1437E-02 |
| Olr670 | olfactory receptor 670 | chr3 | -2,7738 | 8,4800E-04 |
| Arhgap24 | Rho GTPase activating protein 24 | chr14 | -2,7458 | 4,7886E-03 |
| Chmp4bl1 | chromatin modifying protein 4B-like 1 | chr4 | -2,6893 | 1,8182E-02 |
| Cyp2c22 | cytochrome P450, family 2, subfamily c, polypeptide 22 | chr1 | -2,6831 | 1,8353E-02 |
| Oas1k | 2 ' -5 ' oligoadenylate synthetase 1K | chr12 | -2,6817 | 3,8346E-02 |
| Bhlhe41 | basic helix-loop-helix family, member e41 | chr4 | -2,6492 | 1,7613E-03 |
| Vom2r19 | vomeronasal 2 receptor, 19 | chr1 | -2,5430 | 5,2732E-03 |
| Per2 | period homolog 2 (Drosophila) | chr9 | -2,5278 | 2,4173E-03 |
| Epm2a | epilepsy, progressive myoclonus type 2A | chr1 | -2,5177 | 2,6197E-02 |
| Olr624 | olfactory receptor 624 | chr3 | -2,3924 | 5,0065E-03 |
| Epm2a | epilepsy, progressive myoclonus type 2A | chr1 | -2,3916 | 6,0200E-05 |
| Fam46b | family with sequence similarity 46, member B | chr5 | -2,3796 | 5,8007E-03 |
| Dsg2 | desmoglein 2 | chr18 | -2,3392 | 3,0419E-03 |
| Tef | thyrotrophic embryonic factor | chr7 | -2,3233 | 1,3424E-03 |
| Npc1l1 | NPC1 (Niemann-Pick disease, type C1, gene)-like 1 | chr14 | -2,3057 | 1,7134E-02 |
| Znf498 | zinc finger protein 498 | chr12 | -2,2773 | 1,1098E-02 |
| Oas1a | 2'-5' oligoadenylate synthetase 1A | chr12 | -2,2552 | 4,2302E-02 |
| Kif6 | kinesin family member 6 | chr9 | -2,2416 | 3,3620E-03 |

Table S8. The top 20 up and top 20 down regulated genes in rat male CP.

Differential gene expression between sham and gonadectomized rat male CP.

The genes were ranked on their fold changes and the twenty with the highest or lowest fold changes are shown here.
